# Urgent Surgery

**Published:** 1910-12

**Authors:** 


**Urgent Surgery.** By FÉLIX LEJARS. Translated from the Sixth French Edition by WILLIAM S. DICKIE, F.R.C.S. Vol. I. Pp. v, 580. Bristol: John Wright & Sons Ltd. 1910.

The high praise we have given to the first volume of this work can be accorded equally to the second. It is a beautiful exposition of surgical art characterised by marked individuality. The illustrations are particularly fine, although we are more accustomed to gloved operators who are either beardless or masked. Attention may be drawn in particular to the chapters on herniæ, fractures and dislocations, which are especially good and contain many suggestions for the thoughtful surgeon.

In view of the prominence given to massage in the treatment of fracture, it may be well to quote the author: "Nowadays it is recognised that there is quite a group of fractures which ought to be treated exclusively by massage. . . . The general rule is to restrict the stage of immobilisation to the shortest time necessary for the important process of consolidation; it is none the less true that every fracture which is displaced or susceptible of displacement ought forthwith to be reduced and immobilised."

It is of interest to note that Lejars employs Reverdin's needle very generally, even for intestinal suture; and that his description of fractures at the elbow-joint is more consistent than is usually found in text-books. There are few surgeons who would not gain much by the study of this praiseworthy effort of a French surgeon, who has so well succeeded in his intention to write what he has lived, and who has presented the profession with a book of valuable experience, artistically illustrated and pleasurable to read.

